# The Emergence of Complexity: Lessons from DNA

**DOI:** 10.1371/journal.pbio.0020431

**Published:** 2004-12-14

**Authors:** Chengde Mao

## Abstract

The same molecular qualities that endowed DNA with its capacity to carry hereditary information make it a powerful tool to explore the self-assembly of complex nanostructures

How did life emerge from a soup of chemicals? How do patterns such as schools of fish form from individuals? How do voting patterns emerge? Such a diverse array of problems seems completely unrelated. However, they all involve “emergence of complexity.” When individuals come together, they form patterns, structures, and organizations that cannot be discerned from the individuals alone. The study of the emergence of complexity is one of the most active and important areas of research. It is important not only for understanding nature, but also for technological applications, including the fabrication of large-scale integrated nanocircuits using a bottom-up approach, and the preparation of multifunctional “smart” nanomaterials.

DNA evolved to be the primary carrier of genetic information because of its extraordinary chemical properties. These same properties also make DNA an excellent system for the study of self-assembly and self-organization. Two complementary molecules of single-stranded DNA have paired bases that bond with each other and form the well-known double helix structure. Two molecules of double-stranded DNA (duplexes) can further associate together if they have complementary single-stranded overhangs (sticky ends). Intermolecular interactions can be precisely predicted by Watson–Crick basepairing (adenine to thymidine and guanine to cytosine). And, these interactions are structurally understood at the atomic level. Given the diversity of the DNA sequences, we can easily engineer a large number of pairs of DNA duplexes that associate with each other with sequence specificity and in a well-defined fashion. This property is not common among other molecular systems. Small organic and inorganic molecular pairs can interact with each other with specificity and in well-defined structures, but the number of such pairs is limited and their chemistry varies greatly. Protein molecules, such as antibody–antigen pairs, have great diversity and high specificity. However, it is extremely difficult, if not impossible, to predict how proteins interact with each other. In contrast, DNA as a molecular system fulfills all the aforementioned criteria.

In nature, DNA occurs predominantly as a linear molecule, and if its conformations were limited to linearity, it would not be very useful for studying self-assembly. Fortunately, branched DNA structures can be engineered. Holliday junctions, for example, are intermediates that occur during genetic recombination. To model Holliday junctions, a stable four-arm junction has been constructed in which, by design, no two strands are fully complementary to each other (Kallenback et al. 1983; Seeman 2003) (Figure 1A). For example, the 5′ half of strand 2 is complementary to the 3′ half of strand 1, but the 3′ half of strand 2 is complementary to the 5′ half of strand 3 instead of that of strand 1. Combining branched structures and the excellent molecular recognition of DNA, we are ready to engineer complicated DNA nanostructures and use them for studying self-assembly.

**Figure 1:**
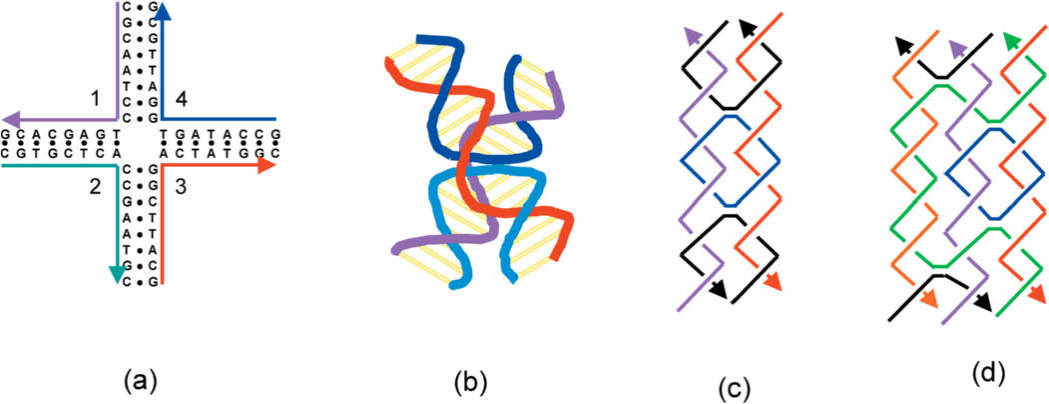
Basic DNA Structures for Self-Assembly (A) A four-arm junction and (B) its three-dimensional structure; (C) a DNA DX; and (D) a DNA TX.

Extensive studies have shown that the four-arm junction adopts an X-shape structure (Figure 1B) under physiological conditions, and the angle between its two helical domains can vary widely (Lilley 2000). It is impossible to construct well-defined large structures from flexible components. To overcome this problem, several well-behaved DNA motifs have been engineered. Double crossover (DX) (Fu and Seeman 1993) and triple crossover (TX) (LaBean et al. 2000) molecules are two early examples (Figure 1C and 1D). In such molecules, two or three DNA duplexes lie side by side. Two neighboring duplexes are joined by two crossovers, which prevent any duplex from twisting against its neighbor duplex. Thus, the interhelical angles become fixed at 0°. Other motifs quickly followed, including the paranemic crossover motif (Shen et al. 2004), rhombus/parallelogram motif (Mao et al. 1999), cross motif (Yan et al. 2003), and several triangle motifs (Chelyapov et al. 2004; Ding et al. 2004; Liu et al. 2004). They all are stable, rigid, and readily designed for self-assembly.

One simple example of self-assembly is the formation of two-dimensional (2D) periodic arrays or 2D crystals. This is also one of the greatest successes in the field of DNA self-assembly (Figure 2). The first 2D DNA crystals were assembled from DX motifs (Winfree et al. 1998). In a 2D crystal, each DX molecule contains four sticky ends (A–A′ and B–B′) distributed on its two component duplexes. The complementarity of the sticky ends is designed in such a way that a DX molecule will interact with another four DX molecules through its four sticky ends. Any two DX molecules can interact with each other though only one pair of sticky ends. Any pair of sticky end interactions will position the two DX molecules in a conformation such that no other sticky ends from these two molecules are in sufficient proximity to interact. As a result of this design, regularly ordered 2D arrays have formed (Figure 2). Following similar strategies, others have designed DNA motifs to assemble into 2D arrays, whose symmetries include tetragonal (Yan et al. 2003), pseudohexagonal (Mao et al. 1999; Liu et al. 2004), and hexagonal (Chelyapov el al. 2004; Ding et al. 2004).

**Figure 2:**
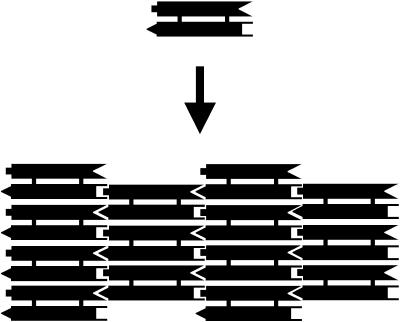
Self-Assembly of a 2D DX Array Each rod represents a DNA duplex. The geometric complementarity represents the sequence complementarity of sticky ends.

Inspired by early theoretical suggestions (Winfree 1998), experimental exploration of aperiodic self-assembly immediately followed. One study applied algorithmic self-assembly to TX molecules (Figure 3) (Mao et al. 2000). The assembling rule “exclusive OR” (XOR) is encoded in the TX molecules. Consider the value of all inputs and outputs as either 1 or 0. For XOR operations, if two inputs are the same, the output will be 0; otherwise, the output will be 1. If molecules X and Y are the input and output, respectively, Y_i_ molecule takes the input from the X_i_ and Y_i−1_ molecules. In other words, the values of the X_i_ and Y_i−1_ molecules determine what Y molecule will be incorporated. There are four different types of Y molecules, whose inputs are (1, 1), (1, 0), (0, 1), and (0, 0). These four, and only four, Y molecules are enough to satisfy any input combination. Two C molecules connect the input and output molecules, which is necessary for the characterization but not essential for the self-assembly process. Sticky ends between the X molecules and the C molecules are longer than those between Y molecules and between Y and X or C molecules. Thus, the C and X molecules assemble first to form the inputs because the association between longer sticky ends is more stable than those between the shorter ones. Then the output Y molecules assemble to the assembled C and X molecules. In that study (Mao et al. 2000), two different input combinations were used, and one of them is shown in Figure 3. The resulting DNA structures are periodic with respect to the backbones, but they are aperiodic in their sequences. Though the resulting four-byte one-dimensional (1D) structures are quite simple, this study demonstrated that aperiodic structures are achievable through self-assembly.

**Figure 3:**
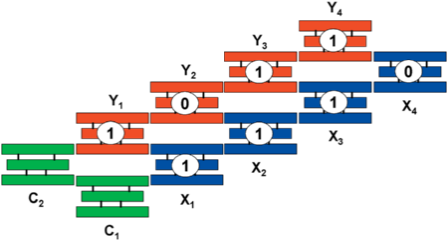
Self-Assembly of a 1D Aperiodic TX Array Based on XOR Operation The value of any input or output is binary, either 1 or 0. If two inputs are the same, the output is 0; otherwise, the output is 1.

Winfree and co-workers in this issue of *PLoS Biology* have extended the algorithmic self-assembly strategy from 1D to 2D (Rothemund et al. 2004). This achievement is certainly a milestone in the field of self-assembly. It overcomes a great challenge, as the structural complexity dramatically increases from 1D to 2D structures. These researchers have applied the same XOR algorithms to DX molecules in their study and achieved fractal structures, Sierpinski triangles (Figure 4). External inputs are in the bottom row. Each row takes inputs from the row immediately below, and sends the operation outputs to the row immediately above. Each position takes two inputs (identical or non-identical) from lower left and lower right positions, and sends identical output to both upper left and upper right positions. The arrows indicate the direction of information flow, or assembly sequences. In their experiment, the rules are encoded in DX molecules. This study is conceptually straightforward, but the experimental challenges are tremendous. One key challenge is assembly fidelity. The right molecules have to compete with partially matched molecules. The concentrations of the competing molecules further complicate the fidelity issue, as some molecules could be rapidly depleted from the solution. In that sense, the current work is quite stunning even though the assembly is far from perfect.

**Figure 4:**
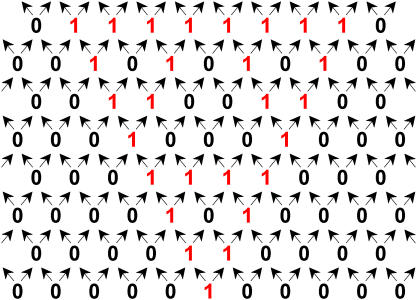
Schematic Representation of Self-Assembly of a Sierpinski Triangle Based on XOR Operation The values in the bottom row are the inputs.

In principle, a wide range of 2D patterns could be generated with the same set of molecules and the same strategy, changing only the first row of the assembly, which specifies the external inputs. Realization of this goal will critically rely on the elimination of assembly errors, or the introduction of error corrections (Winfree and Bekbolatov 2004).

The current work represents a neat approach to understanding the emergence of complexity. It integrates both simulation and wet chemistry. It also provides a plausible approach to nanofabrications. Over the last decade, a variety of methods have been developed, which use biomacromolecules as templates to fabricate nanostructures (Braun el al. 1998; Douglas and Young 1998; Mucic et al. 1998; Fu et al. 2004). Limited by the complexity of the available biomacromolecular templates, simple nanostructures are the usual result: mostly nanowires, nanoparticles, and simple aggregates of nanoparticles. The current work illustrates the possibility of generating more complicated structures and promises unprecedented structural complexity for nanomaterials.

## Abbreviations

1D: one dimensional
2D: two dimensional
DX: double crossover
TX: triple crossover
XOR: exclusive OR
